# Annual Announcement of Lectures in the Atlanta Medical College

**Published:** 1861-10

**Authors:** 


In issuing this announcement of the eighth Course of Lectures in the Atlanta Medical College, the Faculty are not unmindful of the national difficulties which surround them, nor of their duties to the Government in a military point of view, yet, in response to the various inquiries in regard to the probabilities of a course of Lectures in 1862, they have determined to invite Medical Students to the ensuing course, commencing on the first Monday in May next; and ending the last of August. The course will be complete in every particular, as heretofore; and those inclined to pursue the study of Medicine in all its branches, will have the facilities here to do so profitably. Not only will the seven chairs be filled regularly and promptly, but the department of Practical Anatomy so very desirable will be fully supplied with subjects and receive all the attention heretofore bestowed upon it.

### REQUISITES FOR GRADUATION.

The applicant for the Degree must be twenty-one years old, and of good moral character.

He must have attended two full Courses of Lectures in this Institution, or one in this and one in some other respectable School of Medicine, the last of which must be in this Institution.

The evidence of having taken a Course of Dissecting under the direction of a Demonstrator of Anatomy, is also required.

He must have been engaged in the study of medicine the usual time required by other accredited Medical Colleges, under the instruction of a competent physician. A Thesis, of his own composition, on some medical subject, must be presented to the Dean of the Faculty by the first day of August, accompanied with the Diploma Fee.

The Faculty reserve the right, in all instances, to revoke the Degree whenever satisfactory evidence is produced of irregular and unprofessional practices.

### BENEFICIARY FOUNDATION.

In compliance with the terms of an Act passed by the State Legislature, appropriating money to the Atlanta Medical College, one Student from each Congressional District, will be admitted to instruction in the Institution free of charge, upon the presentation of a certificate of appointment from the Representative in Congress from the Congressional District in which he resides.

### FEES.

| | |
|---|---|
| For the Course of Lectures, | $105 |
| Matriculation, (taken only once,) | 5 |
| Dissecting Ticket, (required once only,) | 10 |
| Graduation, | 25 |

### Catalogue of Matriculates--Session 1861.

| *Names.* | *Post Office.* | *Preceptor.* |
|---|---|---|
| Aderhold, H. D. | Carnesville, Ga. | Jessee Low. |
| Almond, J. T. | Conyers', Ga. | S. H. Dean. |
| Arnold, W. E. | Lisbon, La. | Sam'l Turner. |
| Baker, E. J. P. | Taylor's Creek, Ga. | J. P. Mill. |
| Baker, T. H. | Pine Log, Ga. | Western & Hardy. |
| Barr, W. N. | Lexington, S. C. | J. R. Gantt. |
| Bell, Henry Bibb | Elberton, Ga. | J. B. Bell. |
| Boggs, J. N. | Selma, Ala. | James Kent. |
| Bomar, W. T. | Buchanan, Ga. | T. J. Foster. |
| Borders, J. M. | Hamilton, Ga. | W. W. Bruce. |
| Bowen, A. H. | Camden, S. C. | Logan & Powell. |
| Boyd, W. B. | Warrenton, S. C. | J. H. Walton. |
| Brian, T. N. | Gillsville, Ga. | J. D. Long. |
| Bridges, W, P. | Hamilton, Ga. | W. W. Bruce. |
| Brown, J. N | Sand Hill, Ga. | ——— ——— |
| Bryant, D. H. | Woodville, Miss. | F. R. Paddock. |
| Butler, ——— M. D. | Australia, C. A. | University of N. Y. |
| Chisholm, T. B. | Savannah, Ga. | J. J. West. |
| Clawer, Jno. T. | Milltown, Ga. | P. F. Hoyle, |
| Crawley, J. L. | Barnesville, Ga. | R. B. Gardner. |
| Cunningham, L. S., M. D. | Big Creek, Ga. | Medical College, Ga. |
| Davis, Isaiah | Atlanta, Ga. | J. G. Westmoreland. |
| Dennis, F. M. | LaGrange, Ga. | Baugh & Pitman. |
| Dozier. G. R. | Monticello, Ga. | W. D. Maddox. |
| Dozier, M. E. | Atlanta, Ga. | T. H. Dozier. |
| Elder, A B | Watkinsville, Ga. | Lumpkin & Harden. |
| Emery, S C | Jacksonville, Fla. | Holmes Steele. |
| Ezell, W R | Gholsenville, Va. | B J Hicks. |
| Fannin, J B | Troy, Ala. | Practitioner. |
| Florence, S C | Powder Springs, Ga. | J F Cotton. |
| Geer, P F M | Lumber City, Ga. | J T Mann. |
| Gresham, Broussais | Griffin, Ga. | J F Knott. |
| Griffin, G W | Girard, Ga. | D S Perkins. |
| Halbrook, J T | Bold Spring, Ga. | —— —— |
| Hester, E P | Lisbon, La. | T. H Pennington. |
| Johnson, L S | Uchee, Ala. | J Y M Puckett. |
| Johnson, W H | Central Institute, Ala. | B T Smith. |
| Jones, H C | Atlanta, Ga. | J W Jones. |
| Kennedy, Jno S | Midville, Ga. | Wm Houser. |
| Lee, Jno W | Rocky Plains, Ga. | E O Huson. |
| Lynch, James | Atlanta, Ga. | J G Westmoreland. |
| Madden, J M | New Port, Fla. | E M Mattaner. |
| Mangum, W L | Rough & Ready, Ga. | Eli Griffin. |
| McCool, W L | Atlanta, Ga. | J G Westmoreland. |
| McGaughlin. J M | Cedar Grove, Ala. | Robertson & Freeman. |
| McKeynolds, L D | Gaylesville, Ala. | A M McWhorter. |
| Morgan D C | Decatur, Ga. | P F Hoyle. |
| Nash, Chas T | Jefferson, Ga. | W B J Hardman. |
| Nichols, W L | Washington, Ga. | H Andrews. |
| Orsborn, N C | Carnesville, Ga. | D O Orsborn. |
| Palmer, B B | Hopeful, Ga. | E J Palmer. |
| Price, J H | Atlanta, Ga. | J W Price. |
| Powell, J M | Atlanta, Ga. | T S Powell. |
| Roark, M J | Atlanta, Ga. | J G & W F Westmoreland. |

| *Names.* | *Post Office* | *Preceptor.* |
|---|---|---|
| Roberts, W G. | Cedar Branch, Ga. | J T Deavenport. |
| Rogers, J A | Nixbury, Ala. | S L Lennard. |
| Scaife, W L | Homer, La. | J M Scaife. |
| Seago, B L | Linden, Texas. | A J Oliver. |
| Sewell, Jno K | Franklin Springs, Ga. | M. T Alexander. |
| Shirley, E L | China Grove, Ala. | Cook & Powell. |
| Simmons, D A | Townville, S. C. | O R Hooton. |
| Smith, H J | Macon, Ga. | W S Lightfoot. |
| Smith, M P | Trader's Hill, Ga. | ——— ——— |
| Spencer, W O | Jonesboro, Ala. | James Hanghey. |
| Stallings, Jno M | Griffin, Ga. | W B Couch. |
| Shaffer, A J, M D | Lawrenceville, Ga. | Atlanta Med. College. |
| Taylor, G W | Homer, La. | W M P Mallett. |
| Taylor, J P | Homer, La. | M A Cornelius. |
| Thornburgh, Amos | Cassandra, Ga. | J C Lee. |
| Touchstone, W H | Griffin, Ga. | W B Couch. |
| Trice, G W | Louisa C H, Va. | University of Va. |
| Wells, Wm B | Villanow, Ga. | A Clements. |
| Welsh, C C | Butler, S. C. | J H Blair. |
| Willson, Pleasant | Madison, Ga. | Crawford & Knight. |
| Yarbrough, J D | Griffin, Ga. | ——— ——— |

### GRADUATES FOR 1861.

Aderhold, A. D—Georgia: Metritis.
Arnold, W. E—Arkansas: Practitioneer of Medicine.
Boggs, J. N—Alabama: Inflammation.
Boyd, W. B—South Carolina: Digestion.
Bowen, R. H—South Carolina: Hyoscyamus.
Brian, T. N—Georgia: Yellow Fever.
Chisholm, T. B—Georgia: Medicine.
Clower, Jno. T—Georgia: Gunshot Wounds.
Dennis, F. M—Georgia: Effects of Stranguary.
Dozier, M. E—Georgia: Water.
Dozier, G. R—Georgia: Anæsthetics.
Elder, A. B—Georgia: Anthrax.
Emery, L. C—Florida: Yellow Fever.
Ezell, W. B—Virginia: Syphilis.
Gresham, Broussais—Georgia: Symptomatology.
Hester, E. P—Louisiana: Scarlatina.
McReynolds, L. D—Alabama: Changes of Food in Alimentary Canal.
Lee, Jno. W—Georgia: Pneumonia.
Nichols, W. L—Georgia: Digestion.
Powell, J. M—Georgia: Medical Delusions.
Price, J. H—Georgia: History of Medicine.
Scaife, W. L—Louisiana: Typhoid Fever.
Sewell, J. K—Georgia: Asthma.
Stallings, Jno. M—Georgia: Intermittent Fever.
Smith, Henry J—Georgia: Intermittent Fever.
Spencer, W. O—Alabama: Typhoid Fever.
Taylor, J. P—Louisiana: Signs of Pregnancy.
Thornburgh, Amos—Georgia: Theory and Practice of Medicine.
Touchstone, W. H—Georgia:—Signs of Pregnancy.
Welsh, C. C—South Carolina: Colitis.
Willson, Pleasant—Georgia: Menstruation:
32 = Yarbrough, J. D—Georgia: Typhoid Fever.

